# Collaboration and Governance in Integrated Care Systems: A Moroccan Perspective on Lessons From England’s ICS: Comment on "New Ways of Working to Manage and Improve Quality in Integrated Care Systems in England"

**DOI:** 10.34172/ijhpm.9299

**Published:** 2025-11-11

**Authors:** Abdeslam Baalla, Tarik Jellouli

**Affiliations:** Interdisciplinary Research Laboratory in Economics, Finance and Organizational Management, Faculty of Law, Economic and Social Sciences, Sidi Mohamed Ben Abdellah University of Fez, Fez, Morocco.

**Keywords:** Integrated Care Systems, Health Coverage, Territorial Health Groups (GST), Health Governance, Health Inequalities, Morocco

## Abstract

Lalani et al explores how integrated care systems (ICSs) in England are redefining quality management through collaboration, co-production, and a focus on population health. This commentary examines the relevance of these principles for Morocco, where universal health coverage (UHC), the expansion of social protection, and Territorial Health Groups (GSTs, *Groupements Sanitaire Territoriaux*) aim to improve care coordination and reduce health inequalities. ICS-inspired strategies could strengthen Moroccan reforms by promoting decentralized governance, community engagement, and co-production, with GSTs serving as regional coordination platforms. However, financial constraints, centralized institutions, and political pressures to prioritize visible outcomes require local adaptation. Strengthening regional quality committees, addressing social determinants, and empowering community health workers (CHWs) could help align Morocco’s reforms with ICS principles.

## Introduction

 The article by Lalani et al, published in the *International Journal of Health Policy and Management*, investigates how England’s integrated care systems (ICSs), established in July 2022, are redefining quality management through collaborative governance, co-production, and a focus on population health and health inequalities.^1^ These systems aim to shift from hierarchical, assurance-focused models to improvement-driven, system-wide approaches fostering partnerships across health, social care, and local communities. This shift is highly relevant for Morocco, where health system reforms, notably the universal health coverage (UHC) program launched in 2021 and the generalization of social protection, aim to improve care coordination and reduce disparities.^2,3^ Additionally, the implementation of Territorial Health Groups (GSTs,*Groupements Sanitaire Territoriaux*) seeks to enhance regional health management and decentralization.

 Morocco’s health system faces challenges similar to those described in England, including fragmented services, urban-rural disparities, and pressure to address immediate healthcare demands (eg, hospital access) while pursuing long-term goals like health equity.^4^ This commentary examines the applicability of ICS strategies to Morocco, focusing on governance, population health, co-production, and the role of social protection and GSTs in supporting these reforms. It argues that while England’s ICS model offers valuable lessons, Morocco must adapt these approaches to its resource-constrained context and centralized institutional framework.

## Main Arguments

###  Collaborative Governance for Quality Management

 Lalani et al highlight the establishment of new governance structures in ICSs, such as Integrated Care Board Quality Committees and System Quality Groups, to balance quality assurance with improvement. These structures facilitate system-wide learning and collaboration among diverse stakeholders, including the National Health Service, Local Authorities, and community organizations. In Morocco, health governance is fragmented, with the Ministry of Health and Social Protection overseeing healthcare delivery, while social services and public health are managed by regional authorities.^5^ This separation mirrors the pre-ICS challenges in England, where Clinical Commissioning Groups operated in silos.

 Morocco’s UHC program, supported by the generalization of social protection, seeks to integrate primary,^6^ secondary, and tertiary care, while GSTs aim to decentralize health service management at the regional level. However, governance remains centralized, limiting local adaptability. The ICS model suggests that Morocco could benefit from decentralized, collaborative governance structures involving regional health authorities, local governments, and civil society, with GSTs serving as platforms for regional coordination. For instance, GSTs could function as regional quality committees akin to ICS System Quality Groups, fostering shared accountability and aligning health and social care priorities. However, Morocco’s limited financial resources, workforce shortages (eg, a doctor-to-population ratio of 0.74 per 1000 compared to England’s 2.8), and political pressures to prioritize visible outcomes, such as hospital infrastructure, pose significant barriers.^7^

###  Population Health and Health Inequalities

 A key ICS objective is improving population health and reducing inequalities, moving beyond traditional clinical quality metrics to include equity and access, as exemplified by the Core20PLUS5 framework targeting the most deprived 20% of the population.^1^ In Morocco, health inequalities are stark, with 30% of rural Moroccans living over 10 km from a health center.^7^ The UHC program, bolstered by social protection initiatives, aims to address these disparities through expanded insurance coverage and primary care strengthening, while the establishment of GSTs enhance resource allocation at the regional level.^8^ However, progress is slow Owing to funding constraints and a focus on curative rather than preventive care.

 The ICS emphasis on upstream interventions and population health analytics could guide Morocco’s efforts. For example, Morocco could adopt data-driven approaches to identify at-risk populations, with GSTs coordinating these efforts regionally, similar to ICS dashboards. However, challenges in data integration due to disparate health information systems persist.^9^

 Partnerships with non-health sectors (eg, education, housing), supported by social protection programs, could mirror ICS collaborations to address social determinants of health, such as sanitation and nutrition, which significantly impact Morocco’s rural populations. Competing priorities, such as hospital waiting lists, may undermine long-term population health goals, a challenge also evident in Morocco’s politically driven focus on immediate healthcare access.

###  Co-production and Community Engagement

 Co-production, involving patients and communities in service design, is a cornerstone of quality improvement in ICSs but remains underdeveloped due to challenges in ensuring representative participation. In Morocco, community engagement in health policy is limited, often reduced to tokenistic consultations through local associations.^2,4^ Linguistic and cultural diversity, notably among Amazigh and Arabic-speaking communities, complicates inclusive engagement. The ICS approach, relying on organizations like Healthwatch, offers a relevant model for Morocco. Pilot initiatives under UHC and social protection programs have deployed approximately 1000–1170 community health workers (CHWs) in priority regions (2019–2024). These CHWs play a key role in directing populations to maternal, child, and community health services. Integrating them into GSTs could enhance community feedback, improve care coordination, and support local co-production in line with ICS principles.^10^

 However, Morocco’s limited infrastructure for systematic feedback and top-down policy approach impedes effective co-production. Adopting ICS-inspired place-based partnerships, supported by GSTs, could foster equitable collaboration by empowering local health committees to include marginalized groups, such as rural women and low-income populations. This requires investment in training CHWs in participatory methods and developing community platforms.

###  Contextual Adaptation and Implementation Strategies

 The ICS model demonstrates the value of trust-based and collaborative relationships over hierarchical structures. In Morocco, bureaucratic inertia and limited trust between central and regional authorities often complicate coordination. The GSTs, piloted in the Tanger–Tetouan–Al Hoceima region, provide a promising framework for localized collaboration, where regular workshops bring together local health officials, health professionals, and community actors to foster trust and align health priorities according to local needs. Social protection programs, including the generalization of health coverage, can further support these collaborative efforts; However, political pressures and limited resources may slow reform implementation.

###  Structural and Institutional Constraints

 Morocco’s health system remains largely centralized, with limited regional autonomy and fragmented financing and service delivery mechanisms.^11^ Although the generalization of Health coverage has expanded access, gaps in strategic purchasing, regional governance, and financial protection persist.^5,12^ Law 08-22 mandates GSTs to coordinate care, define financing needs, and manage revenue collection; however, operational autonomy and regional leadership capacities are still evolving. Balancing national policy coherence with localized flexibility is essential for ICS principles to take root.

###  Opportunities for Collaborative Governance

 Despite constraints, several opportunities could facilitate the adoption of ICS-inspired practices:

- Public–private partnerships (PPPs): PPPs can mobilize investment and expertise to expand health infrastructure, digital platforms, and workforce training while ensuring accountability and equity.^13,14^
- International and donor support: Morocco benefits from substantial external assistance, including the World Bank’s Health Reform Program and technical support from the World Health Organization (WHO) and the African Development Bank, which contribute to strengthening data integration, health workforce planning, and local governance capacities.^15^
- Empowering local Actors: ICS experiences in England and research in Morocco demonstrate that frontline professionals, “street-level bureaucrats,” and civil society can bridge institutional silos. Integrating these actors into GST planning, monitoring, and evaluation fosters ownership, legitimacy, and trust-based collaboration.^16,17^

###  The GST Pilot: A Learning Platform

 The GST pilot in the Tanger–Tetouan–Al Hoceima region enables Morocco to test and refine integrated governance practices. Key initiatives include the establishment of Regional Quality Committees, Regional Medical Programs, Integrated Health Information Systems, and Multidisciplinary Teams. These efforts are supported by regular stakeholder workshops, where informal discussions enable rapid problem-solving and build trust among local actors, mirroring ICS. These mechanisms aim to coordinate care, pool resources, integrate facilities, monitor patient pathways, and co-produce interventions with communities. This approach, where practice precedes formal policy, mirrors the early experiences of ICS implementation in England.^17^

###  Phased Strategy for Reform

 In the short term (1–2 years), efforts should focus on consolidating the GST pilot and establishing evaluation frameworks supported by public funding and and PPPs.^18^ Over the medium term (3–5 years), expansion to other regions should integrate data systems and CHWs to strengthen coordination and accountability. In the long term (5–10 years), the objective is nationwide scale-up, aligning financing mechanisms and embedding collaborative governance across health and social sectors to ensure sustainable, integrated care.

## Conclusion

 England’s ICSs offer a compelling model for Morocco’s UHC and social protection ambitions, with GSTs serving as a platform for decentralized health management. Morocco’s reform momentum, anchored in the generalization of health coverage, territorial experimentation, and integrated care initiatives, creates favorable conditions for translating ICS principles into practice. Strengthening regional quality committees through GSTs, investing in data integration, and empowering CHWs could further align Morocco’s reforms with ICS strategies.

 However, resource constraints, centralized governance, and political focus on short-term healthcare access necessitate careful adaptation. Policy-makers must balance immediate operational pressures with long-term objectives, including reducing health inequalities and improving overall care quality, drawing on lessons from England that emphasize trust, local empowerment, and iterative learning as essential drivers for system-wide improvement, ultimately guiding Morocco toward a sustainable, integrated, and equitable health system.

## Disclosure of artificial intelligence (AI) use

 Not applicable.

## Ethical issues

 Not applicable.

## Conflicts of interest

 Authors declare that they have no conflicts of interest.
